# SMI-Capsular Fibrosis and Biofilm Dynamics: Molecular Mechanisms, Clinical Implications, and Antimicrobial Approaches

**DOI:** 10.3390/ijms252111675

**Published:** 2024-10-30

**Authors:** Ines Schoberleitner, Michaela Lackner, Débora C. Coraça-Huber, Angela Augustin, Anja Imsirovic, Stephan Sigl, Dolores Wolfram

**Affiliations:** 1 Institute of Pathology, Neuropathology and Molecular Pathology, Medical University of Innsbruck, 6020 Innsbruck, Austria; 2 Department of Plastic, Reconstructive and Aesthetic Surgery, Medical University of Innsbruck, 6020 Innsbruck, Austria; 3 Institute of Hygiene and Medical Microbiology, Medical University of Innsbruck, 6020 Innsbruck, Austria; 4 BIOFILM Lab, Department of Orthopedics and Traumatology, Medical University of Innsbruck, 6020 Innsbruck, Austria; 5 Department of Obstetrics and Gynecology, Medical University of Innsbruck, 6020 Innsbruck, Austria

**Keywords:** silicone mammary implants (SMIs), capsular fibrosis, biofilm formation, foreign body response (FBR), implant surface modification, antimicrobial-impregnated materials, fibrotic response

## Abstract

Silicone mammary implants (SMIs) frequently result in capsular fibrosis, which is marked by the overproduction of fibrous tissue surrounding the implant. This review provides a detailed examination of the molecular and immunological mechanisms driving capsular fibrosis, focusing on the role of foreign body responses (FBRs) and microbial biofilm formation. We investigate how microbial adhesion to implant surfaces and biofilm development contribute to persistent inflammation and fibrotic responses. The review critically evaluates antimicrobial strategies, including preoperative antiseptic protocols and antimicrobial-impregnated materials, designed to mitigate infection and biofilm-related complications. Additionally, advancements in material science, such as surface modifications and antibiotic-impregnated meshes, are discussed for their potential to reduce capsular fibrosis and prevent contracture of the capsule. By integrating molecular insights with clinical applications, this review aims to elucidate the current understanding of SMI-related fibrotic responses and highlight knowledge gaps. The synthesis of these findings aims to guide future research directions of improved antimicrobial interventions and implant materials, ultimately advancing the management of capsular fibrosis and enhancing patient outcomes.

## 1. Introduction

The body’s natural wound-healing process plays a central role in medical implant outcomes [1], particularly in individuals receiving silicone mammary implants (SMIs). When an SMI is placed, the body initiates a wound-healing response that can lead to implant encapsulation and forming a fibrous capsule around the implant, a condition known as capsular fibrosis [1,2]. While this is part of the body’s attempt to isolate the foreign object and restore tissue integrity, excessive fibrosis can result in complications such as pain, implant deformation, and reduced mobility [3,4]. Moreover, the interaction between biofilm formation on the implant surface and the wound-healing process is believed to exacerbate fibrotic responses [5]. Understanding the fundamentals of wound healing is essential for developing strategies to reduce capsular fibrosis and effectively manage biofilm dynamics in individuals undergoing SMI implantation.

### 1.1. Foundations of Wound Healing: Implications for Health and Recovery

Wound healing is a crucial biological process fundamental for restoring the integrity and functionality of damaged tissues. It involves a complex series of overlapping phases: hemostasis, inflammation, proliferation, and remodeling, which are aimed at repairing physical damage, preventing complications, and ensuring overall health and recovery.

Hemostasis is the initial response to tissue damage. When the skin is injured, blood vessels constrict to reduce blood loss, followed by the activation of platelets that form a clot. This clot acts as a temporary barrier against pathogens and provides a scaffold for incoming cells. Platelets release growth factors and cytokines that signal the subsequent phases of healing responses [6,7,8]. The clot is eventually reinforced by the conversion of fibrinogen into fibrin, creating a stable thrombus that aids in tissue repair [6,7,8].

The next phase, inflammation, begins shortly after hemostasis. This phase is marked by the recruitment of immune cells to the wound site to prevent infection and clear debris. Neutrophils are the first responders, arriving within the first 24 h to combat pathogens and release enzymes that help degrade damaged tissue. They are followed by macrophages, which continue the process of debris removal and release cytokines that modulate the inflammatory response [9,10,11,12]. These cells also produce matrix metalloproteinases (MMPs) that degrade extracellular matrix (ECM) components, preparing the wound bed for new tissue formation [10,13]. Macrophages help transition from the inflammatory to the proliferative phase by resolving inflammation and releasing factors that promote tissue repair [14,15].

During the proliferative phase, new tissue is formed to restore the wound area. This includes fibroblast proliferation and ECM deposition, which provide structural support for new tissue. Fibroblasts produce collagen and other ECM components, while endothelial cells contribute to angiogenesis, forming new blood vessels to supply nutrients and oxygen to the growing tissue [16,17,18,19]. Epithelial cells migrate across the wound, re-establishing the skin barrier. This phase also involves wound contraction, driven by myofibroblasts, which helps reduce the wound’s surface area [20,21,22,23,24,25,26].

The final phase, remodeling, focuses on strengthening and refining the newly formed tissue. During remodeling, collagen fibers are realigned and cross-linked to enhance tensile strength. The ECM undergoes continual remodeling by MMPs and their inhibitors, leading to the maturation of the wound tissue [16,24,27,28,29,30]. This phase can last for months to years, during which time the appearance and functionality of the tissue improve as scar tissue is gradually replaced with more normal tissue architecture.

Effective wound healing is essential for repairing the skin’s physical structure and preventing complications such as chronic wounds and pathological scarring, which can lead to persistent pain, impaired function, and increased healthcare costs [9,15]. By ensuring that damaged tissues are effectively repaired, wound healing reduces the risk of further tissue damage and supports the restoration of normal tissue function, ultimately improving outcomes and quality of life for patients.

A key component of wound healing is the prevention of infection. Infections are a common complication in wound care and can significantly impede the healing process, potentially leading to severe health issues [15,31,32,33]. Effective wound healing involves forming a protective barrier through coagulation and subsequent epithelialization, which helps prevent pathogen entry and supports pathogen clearance. This barrier is crucial for minimizing infection risk and promoting a safer, more rapid recovery [15,34].

Another important aspect of effective wound healing is its impact on scar formation. An optimal healing process can reduce excessive scarring, which is vital for both aesthetic and functional reasons [6,7]. Excessive scarring, including hypertrophic scars or keloids, can adversely affect the appearance and function of the repaired tissue. Minimizing scar formation enhances cosmetic outcomes and preserves skin texture and elasticity, which is particularly valuable in surgical and cosmetic procedures [35,36].

Wound healing is also essential for promoting functional recovery. Restoring normal tissue function is crucial for the recovery of movement, sensation, and overall functionality of the affected area [10,37]. In clinical settings, such as after surgery or injury, effective healing ensures that the repaired tissue regains its full functionality, which is critical for enabling patients to return to their normal activities and achieve successful rehabilitation [11].

The overall impact of wound healing extends beyond immediate repair. Chronic wounds or inadequate healing can lead to systemic complications, such as prolonged inflammation, metabolic disturbances, and increased susceptibility to further health issues [29]. Effective wound healing supports overall health by mitigating these risks and reducing the burden on the healthcare system. Promoting rapid and effective repair helps prevent the escalation of health problems and contributes to a more efficient, cost-effective healthcare approach [30].

Advancements in the molecular understanding of wound healing have significantly improved how we approach and manage wounds, leading to notable enhancements in clinical outcomes [20,38]. Key developments include the elucidation of cellular signaling pathways, such as those involving growth factors like Vascular Endothelial Growth Factor (VEGF) and Transforming Growth Factor-beta (TGF-β), as well as cytokines like Interleukin-1 (IL-1) and Tumor Necrosis Factor-alpha (TNF-α) [9,39,40,41]. During wound healing, these pathways regulate crucial processes such as cell proliferation, migration, and differentiation. Understanding these mechanisms has enabled the development of targeted therapies that modulate these pathways to improve healing outcomes.

Research into stem cells has revealed their critical role in wound repair. Mesenchymal stem cells (MSCs) and epithelial stem cells have shown the ability to differentiate into various cell types necessary for tissue regeneration and to secrete factors that promote healing [31]. These advances underscore the potential of stem cell therapies to accelerate wound repair and enhance recovery.

Further progress in understanding the molecular basis of fibrosis has illuminated how excessive scar tissue forms and persists. Key players in this process include fibroblasts, myofibroblasts, and ECM components, along with their regulatory proteins [15,33,34]. This knowledge has facilitated the development of strategies to manage and mitigate excessive scar formation, improving the quality of wound healing.

Recent advancements have provided insights into the mechanisms of inflammation resolution, elucidating how the inflammatory response transitions into the proliferative phase of healing [6]. This includes understanding the roles of anti-inflammatory cytokines and the resolution of inflammatory cell infiltration, which has refined approaches to managing chronic inflammation and supporting effective tissue repair.

Genetic and epigenetic research has identified specific genes and regulatory mechanisms influencing wound healing. Changes in gene expression during various healing phases, along with epigenetic modifications affecting cell behavior, have enabled more personalized treatment approaches [7]. Tailoring interventions based on individual genetic profiles can optimize therapeutic efficacy and improve patient outcomes.

Understanding ECM composition and remodeling has also progressed, revealing how the ECM supports cell migration and tissue repair [14,35,36]. These insights have led to the development of novel biomaterials designed to mimic the natural matrix, enhancing the healing environment and promoting better wound repair.

The field has seen significant advancements with the development of biomaterials such as hydrogels, scaffolds, and nanomaterials. These materials interact with cellular and molecular processes to deliver growth factors, support cell growth, and modulate the healing environment, improving the management of complex wounds [10,11].

Molecular diagnostics and personalized medicine have advanced with the use of biomarkers and genomic profiling, enabling more tailored approaches to wound management [31,42]. This personalization allows for the customization of treatments based on genetic and molecular profiles, enhancing the efficacy of interventions and improving patient outcomes.

Finally, research into immune system interactions with wound healing processes has provided valuable insights into the roles of various immune cells in tissue repair and remodeling [14]. Understanding these interactions has improved the management of inflammatory responses and supported effective healing.

### 1.2. Wound Healing Dynamics, Microbial Biofilms, and Fibrotic Responses in Silicone Mammary Implants

Silicone mammary implants (SMIs) have played a pivotal role in breast surgery since their inception in the early 1960s [43]. Initially developed to meet the demand for cosmetic breast augmentation, these implants quickly became a cornerstone in reconstructive surgery for post-mastectomy patients [44].

Upon the implantation of SMIs, the body initiates an immediate wound response characterized by activation of the innate immune system. Immune cells such as neutrophils, macrophages, and dendritic cells are recruited to the site due to microbial contamination, implant shedding, silicone bleeding, and protein adsorption onto the implant surface [2,45,46,47,48,49,50]. Table 1 provides a comparative overview of these physiological mechanisms and their roles in the wound response to silicone mammary implants.

As the healing progresses, the body forms a fibrous capsule around the implant to isolate the foreign material. This involves the deposition of ECM components such as collagen, leading to the development of a fibrous capsule [46,47,49,51,52]. The extent of fibrosis around silicone mammary implants is shaped by both the initial inflammatory response and the length of chronic inflammation. Persistent inflammation, caused by continuous protein adhesion to the SMI surface and ongoing microbial infiltration, can result in excessive ECM remodeling, increased collagen deposition, and subsequent contracture of the fibrous capsule.

Microbial biofilms that form on the implant surface can persistently stimulate the immune system, leading to prolonged inflammation [53,54,55]. This persistent inflammation is a major factor contributing to excessive ECM remodeling and increased collagen deposition, which exacerbates fibrosis [50,55,56,57]. The continued presence of biofilms intensifies the inflammatory process and promotes the formation of a thick, contracted capsule, ultimately resulting in capsular contracture [58,59,60].

The impact of antimicrobial agents on fibrosis around silicone mammary implants is significant in managing and mitigating fibrotic responses. Antimicrobial treatments, including the use of antimicrobial coatings on implants and systemic antibiotics, aim to prevent or reduce microbial biofilm formation and associated chronic inflammation [61,62,63,64,65].

In examining the management of fibrosis, this review explores the intricate biological pathways involved in the formation and remodeling of fibrous capsules around SMIs. Special attention will be given to how chronic inflammation and excessive ECM remodeling contribute to fibrosis and capsular contracture.

The role of microbial transmission, particularly the formation of biofilms, will be a central focus. Biofilms on implant surfaces can lead to persistent stimulation of the immune system, resulting in prolonged inflammation and exacerbated fibrotic responses. Understanding the relationship between microbial contamination, biofilm formation, and chronic inflammation is essential for developing effective strategies to enhance implant biocompatibility and reduce complications.

Antimicrobial strategies are crucial in controlling infections and improving implant outcomes. Our work assesses current antimicrobial approaches, including antimicrobial coatings and systemic antibiotics, evaluating their effectiveness in preventing or reducing biofilm formation and managing infection. These strategies are vital for reducing the risk of complications and enhancing the long-term success of implants.

Additionally, we discuss novel antiseptic therapies, which offer new opportunities for improving wound healing and reducing infection rates. By examining recent advancements in antiseptic agents, including their mechanisms of action and clinical applications, we aim to identify innovative approaches for enhancing the safety and efficacy of silicone mammary implants.

Through a comprehensive overview of these topics, we want to provide valuable insights into the latest advancements in fibrosis management, infection prevention, and antiseptic therapies. By connecting molecular research with practical clinical applications, we aspire to improve our understanding of these critical factors and their implications in patient care.

## 2. From Wound to Peri-SMI Capsular Fibrosis

Fibrosis is a pathological condition characterized by the excessive accumulation of ECM components, particularly collagen, leading to scar tissue formation and impaired tissue function [2]. This process is often a consequence of chronic inflammation and injury, where normal wound-healing responses become dysregulated, resulting in persistent fibrosis [2,45,66,67].

### 2.1. Immediate Inflammatory Response and Early Fibrosis

#### 2.1.1. Acute Inflammatory Response

Upon silicone implant placement, the body’s initial reaction involves an acute inflammatory response. The implant’s surface, whether smooth or textured, acts as a foreign body, triggering a cascade of immune responses. Macrophages are among the first responders, recognizing and attempting to engulf the silicone material. This process, often leading to impaired phagocytosis, results in prolonged inflammation [2,45]. Macrophages release a range of pro-inflammatory cytokines such as tumor necrosis factor-alpha (TNF-α), interleukin-1β (IL-1β), and interleukin-6 (IL-6), which recruit additional immune cells and activate fibroblasts [12,45,66,68,69].

Fibroblasts, in response to inflammatory cytokines, transition from a quiescent to an activated state, characterized by the secretion of matrix metalloproteinases (MMPs) and cytokines that facilitate tissue remodeling [2,66,70]. Early fibrosis begins with the accumulation of fibroblasts around the implant, contributing to the formation of a provisional matrix. This matrix, rich in collagen type III, serves as a scaffold for subsequent repair processes [52].

#### 2.1.2. Early Fibrotic Changes

The transition from acute inflammation to early fibrosis is marked by the differentiation of fibroblasts into myofibroblasts [71,72], cells with contractile properties due to the expression of α-smooth muscle actin (α-SMA) [1,23,73]. These myofibroblasts are integral to the formation of the fibrous capsule, exerting mechanical forces that influence the organization of the ECM [1]. The initial fibrotic response involves a temporary increase in ECM components such as collagen and fibronectin, which are later replaced by more stable collagen types I and III as the fibrosis matures [46,74,75,76,77].

#### 2.1.3. Molecular Signaling Pathways

Key signaling pathways driving early fibrosis include the transforming growth factor-beta (TGF-β) pathway, which plays a crucial role in fibroblast activation and myofibroblast differentiation [78,79,80]. TGF-β stimulates fibroblasts to produce ECM proteins and to adopt a contractile phenotype. Additionally, the nuclear factor-kappa B (NF-κB) pathway is activated in response to inflammatory cytokines, further promoting fibroblast proliferation and ECM deposition [81].

### 2.2. Chronic Inflammation and Capsular Contracture

#### 2.2.1. Chronic Inflammatory Response

As the inflammatory response persists, chronic inflammation ensues. This phase is characterized by the continued presence of immune cells, particularly macrophages and T cells, which sustain the inflammatory milieu [45]. Macrophages in this stage often differentiate into foreign body giant cells (FBGCs), which are involved in chronic inflammation and fibrosis [69,82,83,84]. Pro-inflammatory cytokines such as TNF-α and IL-1β continue to drive fibroblast activity and ECM deposition [71,72].

#### 2.2.2. Capsular Contracture Formation

The progression to capsular contracture involves a shift from initial fibrosis to a more organized and denser fibrous capsule. This process is marked by increased collagen deposition, particularly collagen type I, and the development of a rigid fibrotic capsule around the implant [76,85]. The contractile activity of myofibroblasts, driven by sustained TGF-β signaling [40,79], contributes significantly to capsule contraction and rigidity [23,29,86].

The Baker classification system, which assesses the severity of capsular contracture, reflects the extent of fibrotic encapsulation and its impact on implant aesthetics and function [87,88,89]. This system categorizes capsular contracture into four distinct grades, reflecting the degree of fibrous tissue formation around the implant and its impact on both the aesthetics and functionality of the breast. In Grade I, the breast appears soft and natural, indicating minimal to no contracture. Grade II describes a breast that is slightly firm but still looks normal, suggesting some fibrous encapsulation is present. Moving to Grade III, the breast becomes firm and may appear abnormal, indicating a significant degree of contracture that can affect the shape and overall appearance. Finally, Grade IV signifies a hard breast with a distorted implant, reflecting severe capsular contracture that often necessitates surgical intervention [87,88,89].

The chronic inflammation and continuous fibroblast activation lead to a thickened capsule, which can impact the implant’s position and patient comfort [4,90].

#### 2.2.3. Chronic Inflammatory Pathways in Sustained Fibroblast Activation and Fibrosis

In chronic inflammation, the persistent activation of TGF-β and NF-κB pathways continues to drive fibroblast activity and ECM remodeling [40,78,79,91]. TGF-β signaling enhances the synthesis of collagen and other ECM proteins [78,79], while NF-κB promotes the expression of inflammatory cytokines and ECM-degrading enzymes like MMPs [81,92]. The interplay between these pathways results in a fibrotic environment that sustains capsule formation and contracture.

#### 2.2.4. Immune Cell Interactions

T cells, particularly CD4+ T helper cells, play a crucial role in chronic inflammation [93]. A predominance of T helper 2 (Th2) and T helper 17 (Th17) cells is associated with increased fibrosis, as these cells secrete cytokines that promote fibrotic responses [94,95]. Regulatory T cells (Tregs) also influence fibrosis, with their presence often inversely proportional to the severity of capsular contracture [93,96,97]. Tregs can modulate the immune response and influence the fibrotic process, though their role can be complex and context-dependent [47].

#### 2.2.5. S100 Proteins on Inflammation and Fibrosis

S100 proteins are a family of calcium-binding proteins that are implicated in various inflammatory and fibrotic processes [98,99,100]. They are involved in regulating cell motility, differentiation, and inflammatory responses [99,100]. S100A8 and S100A9, for instance, are released by activated macrophages and are known to participate in the inflammatory response by promoting neutrophil chemotaxis and activating other inflammatory cells [100,101].

In the context of silicone implants, S100 proteins can contribute to the pathogenesis of capsular fibrosis. Elevated levels of S100A8 and S100A9 have been associated with chronic inflammation and fibrosis in various tissues, including those surrounding implants [46,47,101]. These proteins interact with receptor RAGE (receptor for advanced glycation end-products) on immune cells, modulating cytokine release and fibroblast activation [101,102]. Their presence in the fibrotic capsule can exacerbate inflammatory responses and promote fibroblast proliferation and ECM deposition, thereby influencing the severity of capsular fibrosis [100].

**Figure 1:**
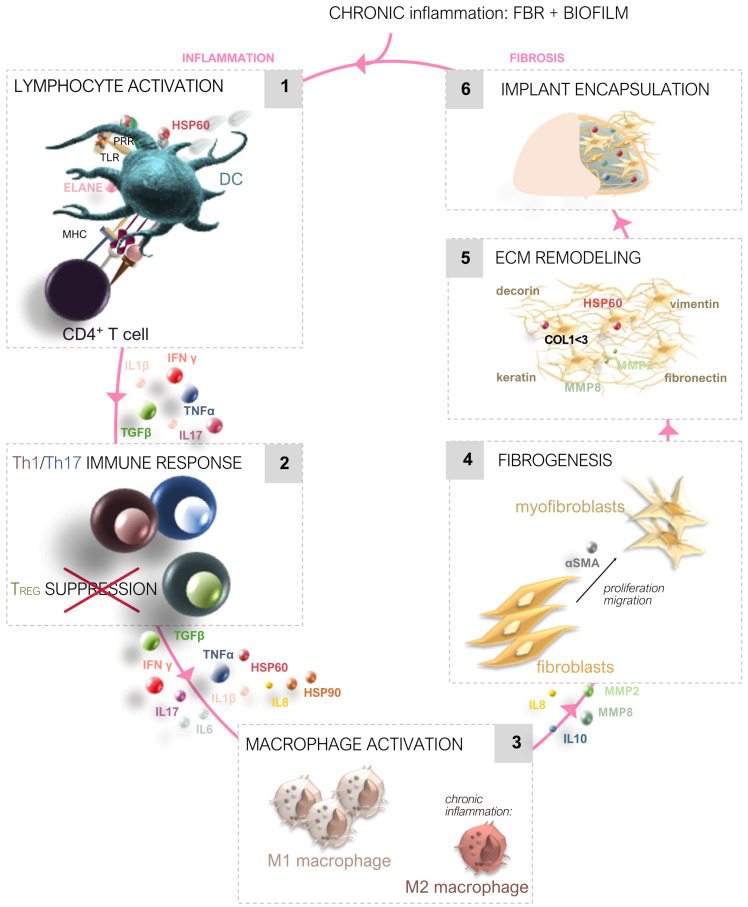
Stages of the inflammatory and fibrotic response SMI insertion, illustrating the transition from immediate post-implantation inflammation to long-term fibrosis and implant encapsulation, with a detailed schematic of the cellular and molecular events driving these processes. Schematic representation of the cellular and molecular events involved in the inflammatory and fibrotic processes: (1) Lymphocyte Activation: CD4+ T cells are activated by dendritic cells (DCs) presenting antigens through major histocompatibility complex (MHC) molecules. Heat shock proteins (HSP60) and other danger-associated molecular patterns (DAMPs) interact with pattern recognition receptors (PRR) and toll-like receptors (TLR), initiating the immune response. (2) Th1/Th17 Immune Response: Activated T cells differentiate into Th1 and Th17 subsets, secreting pro-inflammatory cytokines (IL-1β, IFN-γ, TNF-α, IL-17), which drive the inflammatory response while suppressing regulatory T cells (Treg). (3) Macrophage Activation: M1 macrophages, stimulated by the inflammatory environment, contribute to chronic inflammation. Over time, there may be a transition to M2 macrophages associated with tissue repair and fibrosis. (4) Fibrogenesis: Myofibroblasts arise from fibroblasts under the influence of cytokines like TGF-β, leading to the production of ECM components and the development of fibrotic tissue. (5) ECM Remodeling: The ECM undergoes significant changes, with increased production of proteins such as COL1 < 3, vimentin, and decorin. Matrix metalloproteinases (MMP2, MMP8) are involved in the remodeling process, regulated by factors like HSP60. (6) Implant Encapsulation: The final outcome is the encapsulation of the implant by fibrotic tissue, forming a dense fibrous capsule around the implant due to the accumulation and remodeling of ECM components.

Figure 1 illustrates the complex sequence of events from the immediate inflammatory response leading to the development of fibrosis and eventual capsular contracture following SMI insertion. This figure emphasizes how initial acute inflammation, triggered by tissue damage and the immune system’s response to the foreign body, progresses to chronic inflammation and fibrosis. Over time, these processes contribute to the formation of a dense, fibrotic capsule around the implant, characterized by increased ECM deposition and the activation of fibroblasts and myofibroblasts, ultimately leading to capsular contracture.

### 2.3. Immediate and Chronic Inflammatory Triggers Following SMI Insertion

#### 2.3.1. Immediate Post-Implantation Response

**Figure 2:**
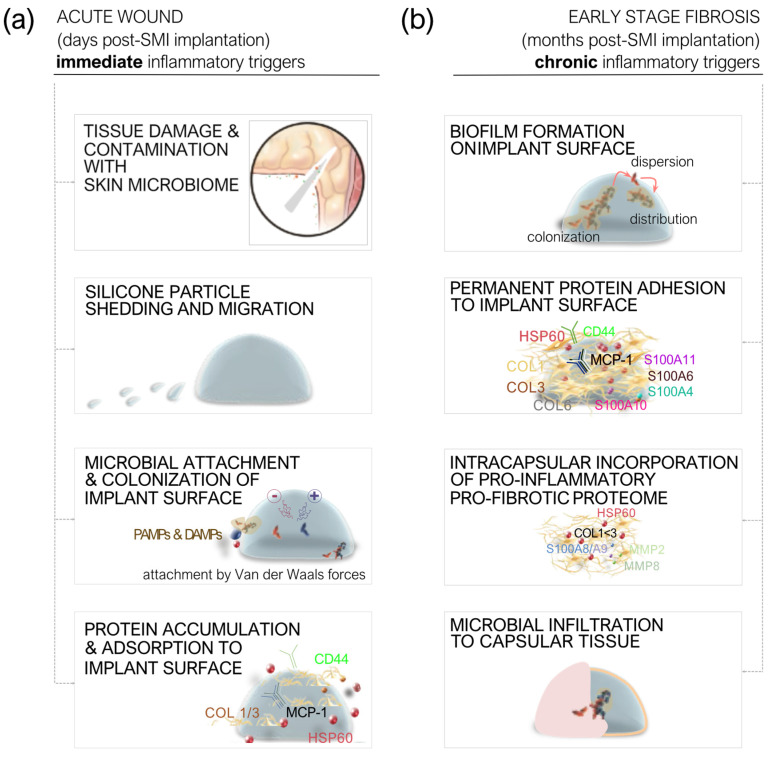
Mechanisms triggering immediate and chronic inflammation in response to silicone mammary implants. (**a**) Immediate Inflammatory Triggers Post-Implantation: The acute inflammatory response is initiated immediately after silicone mammary implant (SMI) insertion due to tissue damage, skin microbiome contamination, and the introduction of DAMPs and PAMPs. Silicone microparticle shedding, microbial attachment, and protein adsorption on the implant surface further intensify the inflammatory response, with mediators like MCP-1, collagen (COL1/3), and CD44 accumulating in the wound environment. (**b**) Chronic Inflammatory Triggers and Early-Stage Fibrosis: Months after implantation, persistent inflammation is driven by biofilm formation on the implant surface and the adhesion of proinflammatory and profibrotic factors. Microbial infiltration into fibrotic tissue and extensive ECM remodeling, involving vimentin, decorin, and fibronectin, result in the development of a dense fibrotic capsule.

Upon the insertion of silicone mammary implants (SMIs), the body mounts an acute inflammatory response (Figure 2a) [2,45,46,47,50,103,104]. This is initiated by factors such as tissue damage, contamination by the skin microbiome, and the introduction of damage-associated molecular patterns (DAMPs) and pathogen-associated molecular patterns (PAMPs) [54,55,105]. The immune system rapidly activates, recruiting immune cells like neutrophils and macrophages to clear the damaged tissue and manage the initial microbial contamination [50,55,57,105]. Additionally, microparticulate silicone may be shed from the implant, migrating into the surrounding tissues and exacerbating the inflammatory response. Microbial attachment to the implant surface, along with the adsorption of wound proteins, triggers further immune activation [50,105]. Inflammatory mediators such as monocyte chemoattractant protein-1 (MCP-1), collagen types I and III (COL1/3), and cellular adhesion markers like CD44 accumulate at the wound site, amplifying the acute response and setting the stage for potential fibrosis [46,50].

#### 2.3.2. Early-Stage Fibrosis (Months Post-Implantation)

As the healing process continues (Figure 2b), a persistent inflammatory state emerges [2,45]. Biofilms can develop on the implant surface, contributing to chronic inflammation due to the ongoing presence of microbial species and their adhesion to the implant [50,105]. This chronic inflammation drives the activation of pro-inflammatory and pro-fibrotic pathways, with these factors becoming incorporated into the developing fibrotic tissue around the implant [46,47]. Over time, the extracellular matrix (ECM) undergoes significant remodeling, marked by the deposition of proteins such as vimentin, decorin, and fibronectin [2,45]. These changes contribute to the formation of a dense fibrotic capsule that surrounds the implant, which is further infiltrated by microbial species, perpetuating the chronic inflammatory state [50]. Ultimately, this sustained inflammation and ECM remodeling lead to the development of capsular fibrosis and contracture around the implant [48,56,58,59,60,106].

### 2.4. Influence of Surface Characteristics on Inflammation and Fibrotic Pathways

#### 2.4.1. Surface Texture and Inflammation

The surface characteristics of silicone implants, such as texture and roughness, significantly influence the inflammatory and fibrotic responses. Textured surfaces tend to provoke a more robust inflammatory response compared to smooth surfaces. Studies have shown that rougher surfaces increase immune cell activation and cytokine secretion, contributing to heightened fibrosis and contracture [47,50,103,104,107].

Surface texture affects fibroblast behavior, including their attachment, proliferation, and differentiation [104]. Textured surfaces often lead to more pronounced fibroblast activation and increased collagen deposition, while smoother surfaces can result in less severe fibrotic responses [47,104]. This variation in fibrosis is linked to differences in cytokine profiles and immune cell interactions, with rougher surfaces promoting a more aggressive inflammatory environment [47].

#### 2.4.2. Surface Characteristics and Cellular Responses

The impact of implant surface characteristics on cellular responses is mediated through various molecular signaling pathways. For instance, rougher surfaces may enhance the activation of NF-κB and TGF-β pathways, leading to increased fibroblast activity and ECM deposition [47]. Surface roughness also influences macrophage polarization, with smoother surfaces promoting an anti-inflammatory M2 macrophage phenotype and rougher surfaces favoring a pro-inflammatory M1 phenotype [47,108,109].

## 3. Microbial Adhesion, Colonization, and Biofilm Formation on SMI

Despite rigorous adherence to sterilization and disinfection protocols [110,111,112,113], SMIs can become sites for microbial adhesion, colonization, and biofilm formation. This occurs despite the inert nature of silicone, which itself lacks antimicrobial properties [114]. The body’s immune response to these implants plays a crucial role in managing microbial threats, but it also inadvertently contributes to a conducive environment for bacterial colonization, particularly when biofilms are formed on the implant surface [53,54,55,59,60,115,116].

### 3.1. Microbial Transmission and Surface Adhesion

During surgical implantation, SMIs may become contaminated through various pathways, including skin microbiota, operating room environments, or surgical instruments. Staphylococcal species, particularly *Staphylococcus epidermidis*, are commonly implicated due to their ubiquitous presence on human skin and the bacterial ability to adhere to the implant surface [50,58,59]. Once these microbes come into contact with the SMI surface, especially during the acute wound phase (24–120 h post-operation), they begin the process of adhesion, which is influenced by the surface characteristics of the implant [50].

Surface topography is a critical determinant of microbial adhesion. Rougher surfaces, characterized by higher surface roughness (average roughness Ra 60 µm), provide more surface area and microenvironments conducive to bacterial adherence [50,117]. This is particularly evident in studies where rougher SMI surfaces attracted more bacteria, including *S. epidermidis*, and facilitated thicker biofilm growth compared to smoother surfaces [50,106,118,119,120,121,122]. The initial phase of microbial adhesion involves reversible attachment, where bacteria adhere to the implant surface through weak van der Waals forces and hydrophobic interactions [123,124]. If conditions are favorable, this progresses to irreversible attachment, where bacteria anchor themselves more securely, often using surface appendages like pili or fimbriae [54,117].

### 3.2. Colonization and Biofilm Formation

Following successful adhesion, the bacteria begin to proliferate and secrete extracellular polymeric substances (EPSs), forming a biofilm [54,117]. Biofilms are complex, structured communities of bacteria encased in a self-produced matrix of EPS, including polysaccharides, proteins, and DNA [54,55,125,126,127]. This matrix helps bacteria adhere more securely to the implant surface and provides protection from the host immune system and antibiotic treatments [1,2,3,4,5]. Biofilm formation is a key factor in the persistence of bacterial infections associated with SMIs, as bacteria within a biofilm can be up to 1000 times more resistant to antibiotics than their planktonic (free-floating) counterparts [54,125].

**Figure 3:**
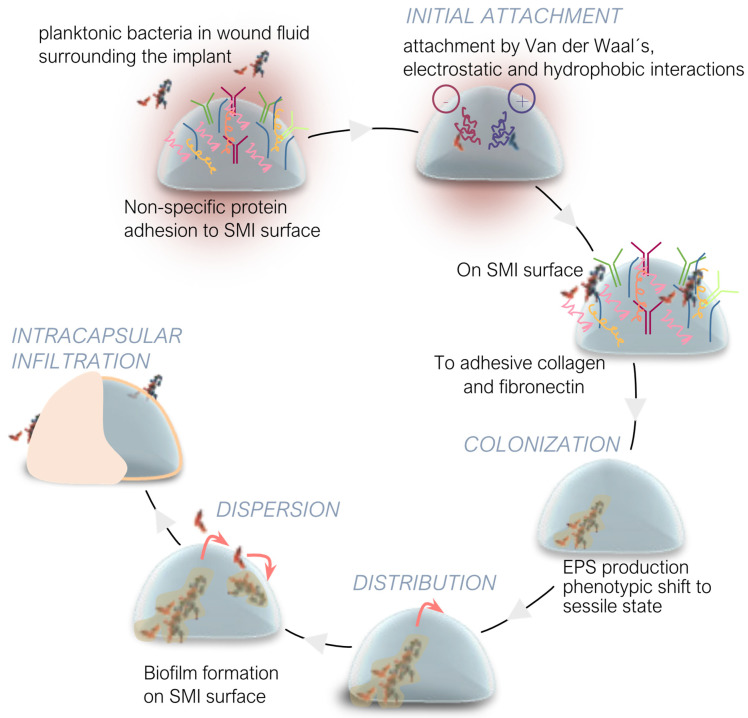
Bacterial contamination and biofilm development contribute to capsular contracture. Microbial colonization during implant placement begins with the transfer of the skin microbiome to the implant surface through surgical tools and incision, embedding the implant into the lower tissue layers. Microbial Proliferation in the Acute Wound: The patient’s skin microbiome is transferred to the implant surface during surgery, via the scalpel, incision, and implant placement. Reversible Phase: In this early stage, planktonic bacteria adhere to the implant surface and the adhesive proteome through electrostatic and hydrophobic forces. At this point, the bacterial adhesion is reversible, and appropriate interventions can still prevent colonization. Irreversible Phase: As time progresses, bacteria establish a firm attachment to the surface, proliferating and forming a biofilm. The biofilm matrix shields the bacteria from the host’s immune system and antimicrobial treatments, promoting a persistent infection. Capsule Formation and Contracture: The persistent presence of biofilms triggers an ongoing antimicrobial inflammatory reaction, contributing to the development of a fibrous capsule around the implant. This process can result in capsular contracture, where the capsule thickens and tightens, causing complications.

Figure 3 illustrates the sequence of events from initial microbial adhesion to biofilm development, ultimately leading to capsular contracture. The transfer of skin microbiota during implant placement, early bacterial colonization, biofilm formation, and the resulting fibrous capsule formation are critical steps in the pathogenesis of implant-associated infections and complications.

The biofilm development on SMIs is particularly concerning because it can lead to chronic infections that are difficult to detect and treat [53,55,56,57,58,60,116,128]. For example, subclinical infections associated with *S. epidermidis* are often asymptomatic but can result in significant complications such as capsular contracture [58,59,129,130]. The chronic nature of these infections is partly due to the immune system’s inability to effectively clear biofilms, as the protective EPS matrix inhibits the penetration of immune cells and antimicrobial peptides (AMPs) [50,59].

## 4. Reducing Capsular Contracture: Antimicrobial Strategies in Breast Implant Surgery

Sterilization and disinfection are crucial components of infection control in healthcare settings, forming the bedrock of patient and healthcare personnel safety [110]. In hospital environments, a multitude of surgical and invasive procedures are performed daily, which inherently introduces the risk of pathogen transmission. The improper sterilization or disinfection of medical instruments can result in severe infection risks, including the transmission of hepatitis B and environmental pathogens [131,132,133,134,135]. To mitigate these risks, healthcare facilities must employ effective decontamination methods for medical devices. Healthcare providers are pivotal in implementing these procedures to reduce and eliminate potential infections [112]. Establishing specific sterilization and disinfection guidelines based on the intended use of medical devices and associated infection risks is mandatory [110]. Given the rise of newly emerging and multidrug-resistant pathogens in healthcare centers, medical professionals need to possess a thorough understanding of these techniques to prevent the spread of infections. The International Organization of Standardization has set standardized definitions and criteria for sterilization, disinfection, and cleaning, with initial guidelines established in 2004 and subsequently updated in 2010 [111,113].

**Figure 4:**
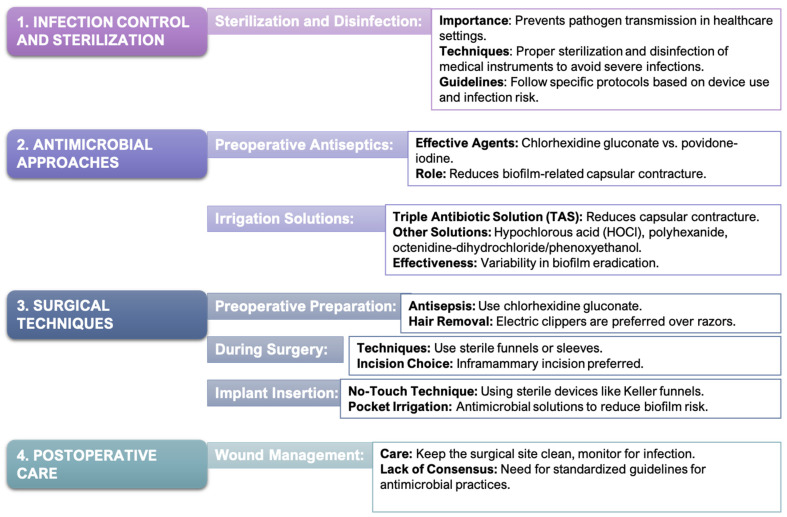
Strategies for Reducing Capsular Contracture in Breast Implant Surgery. Infection Control and Sterilization: Proper sterilization and disinfection of medical instruments are emphasized to prevent pathogen transmission in healthcare settings. Guidelines tailored to the specific use of medical devices and associated infection risks are critical for avoiding severe infections. Antimicrobial Approaches: The effectiveness of preoperative antiseptics, including chlorhexidine gluconate and povidone-iodine, is compared, with a focus on their role in reducing biofilm-related capsular contracture. The use of irrigation solutions such as Triple Antibiotic Solution (TAS), polyhexanide, and hypochlorous acid is also explored for biofilm eradication. Surgical Techniques: Preoperative preparation, including antisepsis methods and hair removal techniques, is combined with intraoperative strategies like the no-touch technique and antimicrobial pocket irrigation to reduce bacterial exposure. The use of sterile devices like Keller funnels and the selection of specific incision types, such as inframammary incisions, are recommended to minimize contamination risks. Postoperative Care: Proper wound management, including keeping the surgical site clean and monitoring for infections, is crucial for preventing complications and ensuring optimal surgical outcomes.

As shown in Figure 4, strategies for reducing capsular contracture in breast implant surgery emphasize the importance of infection control, including sterilization and the use of antimicrobial agents like chlorhexidine gluconate to minimize bacterial contamination. These approaches, combined with surgical techniques like the no-touch method and postoperative care protocols, play a critical role in preventing biofilm formation and ensuring better surgical outcomes.

### 4.1. Antimicrobial Approaches to Mitigate Postoperative Infections and Biofilm Formation

In the context of aesthetic breast surgery, there has been a noted increase in the rate of postoperative surgical site infections (SSIs). The most commonly identified bacteria in these infections include *Staphylococcus epidermidis*, *Staphylococcus aureus*, *Escherichia coli*, *Pseudomonas aeruginosa*, *Propionibacterium acnes*, and *Corynebacterium* species [59,65,136,137,138]. Preoperative skin antiseptics play a critical role in reducing these postoperative complications. While povidone-iodine and other antimicrobial irrigations have been effective in reducing these complications [139], chlorhexidine gluconate has demonstrated superior efficacy in minimizing biofilm-related capsular contracture compared to povidone-iodine [116].

The relationship between bacterial contamination and implant-related complications was first elucidated by Burkhardt et al. in 1986, highlighting the significant impact of local antimicrobial agents in reducing the incidence of capsular contracture in retromammary implants [140]. Betadine pocket irrigation was once the standard practice for preventing such complications [141]; however, in 2000, the FDA contraindicated its use, prompting the adoption of alternative solutions such as triple antibiotic solution (TAS) [142]. TAS has since been associated with a substantially lower rate of capsular contracture in patients undergoing breast augmentation [57,143,144,145,146]. Although TAS is widely favored, some studies suggest that betadine-containing irrigations may be more effective in certain contexts [145,147].

The timing and duration of irrigation solutions have also been revisited, with evidence suggesting that prolonged exposure times may enhance their efficacy [148,149,150,151]. Surveys conducted among members of the American Society of Plastic Surgeons (ASPS) have highlighted the use of 0.1% polyhexanide as a preferred antimicrobial agent during breast augmentation pocket irrigation. This trend underscores its recognition within the plastic surgery community for its effectiveness in reducing bacterial contamination associated with breast prostheses. Nevertheless, there remains significant variability in the antimicrobial techniques and agents preferred by ASPS members, emphasizing the need for standardized guidelines in breast pocket irrigation and implant soaking.

Recent studies have further examined the anti-biofilm efficacy of various irrigation solutions, comparing hypochlorous acid (HOCl), polyhexanide (Lavasorb^®^), and octenidine-dihydrochloride/phenoxyethanol using an innovative in vitro human plasma biofilm model [62]. Their findings revealed that hypochlorous solutions were less effective in eradicating biofilms compared to polyhexanide and octenidine–dihydrochloride/phenoxyethanol, suggesting that HOCl may not be suitable for standalone biofilm eradication [62].

Despite these advances in understanding and technique, staphylococci remain the most common microorganisms in the axillary flora, and antibiotics targeting these bacteria have not significantly impacted the incidence of SSIs [152]. Furthermore, preoperative antibiotic prophylaxis has not markedly reduced SSIs in breast cancer surgery [153,154]. Patient compliance is also critical, as noncompliance with medication regimens can double the risk of infection following breast surgery [155].

The routine application of antimicrobial pocket irrigation, the use of implant soaking agents, and various preventative strategies have been shown to collectively reduce the rates of capsular contracture [156]. However, despite the well-documented association between bacterial contamination and surgical complications, there remains a conspicuous lack of universally accepted, evidence-based guidelines for antimicrobial breast pocket irrigation and perioperative antibiotic administration. Furthermore, the literature provides conflicting evidence regarding the effectiveness of various pocket irrigation solutions, such as HOCl [157], and there is ongoing debate about the role of systemic antibiotics in preventing surgical site infections (SSIs), with some studies suggesting a limited impact.

In conclusion, while significant progress has been made in understanding and addressing SSIs and capsular contracture in breast surgery, there remains a critical need for consensus on antimicrobial practices. The establishment of evidence-based guidelines for antimicrobial irrigation in implant-based breast surgery is essential to optimize patient outcomes and reduce the incidence of complications related to bacterial contamination.

### 4.2. Surgical Techniques for Minimizing Bacterial Contamination

During SMI implantation, minimizing bacterial contamination is crucial for reducing complications such as biofilm-associated fibrotic capsule formation [150,158,159,160]. Effective preoperative preparation plays a vital role in this process. Using electric clippers for hair removal instead of razors minimizes microabrasions and the risk of introducing bacteria into the surgical field [161].

During the surgical procedure, employing a no-touch technique is essential for minimizing the risk of contamination [162]. Devices like Keller funnels facilitate this approach by ensuring that implants are handled in a sterile manner, reducing the chance of bacterial exposure [162].

The technique of implant insertion also plays a significant role in controlling contamination [163]. Using sterile funnels or sleeves during the insertion process limits the implant’s contact with non-sterile surfaces, further reducing bacterial exposure [162,164]. Additionally, the choice of surgical incision is important; the inframammary incision is favored over the periareolar approach due to its lower infection rates and better control over implant exposure to contaminants [163,164,165,166].

Postoperative care is equally critical in minimizing contamination risks. Proper wound care, including keeping the surgical site clean and monitoring for signs of infection, is essential to prevent complications [137,147].

In summary, the choice of surgical incision and careful postoperative care further contribute to minimizing contamination risks and ensuring optimal outcomes.

### 4.3. Translating Preclinical Findings to Clinical Applications

The translation of preclinical findings into clinical applications is critical in the context of antimicrobial strategies aimed at reducing complications in breast implant surgery. Numerous studies have established the effectiveness of various antimicrobial agents in laboratory settings, yet the application of these findings to clinical practice often presents challenges. In aesthetic breast surgery, where there is a rising incidence of postoperative surgical site infections (SSIs), translating these insights effectively is essential for improving patient outcomes.

Preclinical studies have consistently demonstrated that certain antimicrobial agents, including povidone-iodine and chlorhexidine gluconate, can significantly reduce bacterial contamination and biofilm formation around implants. For instance, chlorhexidine gluconate has shown superior efficacy in minimizing biofilm-related capsular contracture compared to povidone-iodine, underscoring its potential for clinical application in surgical protocols [116,139]. Furthermore, the introduction of the triple antibiotic solution (TAS) has been informed by preclinical data indicating its effectiveness in reducing capsular contracture rates in breast augmentation procedures [57,143,144,145,146,147].

Despite these advancements, the implementation of preclinical findings in clinical practice remains inconsistent. A notable example is the variability in the use of antimicrobial agents among practitioners, as highlighted by surveys of the American Society of Plastic Surgeons (ASPS) members. While 0.1% polyhexanide has gained recognition as an effective antimicrobial agent during breast augmentation pocket irrigation, the lack of standardized guidelines leads to significant differences in practice patterns [150]. Such disparities can result in variations in patient outcomes, emphasizing the need for consensus based on robust clinical evidence.

Moreover, the exploration of novel irrigation solutions, such as hypochlorous acid (HOCl), has been approached through preclinical models, yet clinical applicability remains debated. Findings suggest that while HOCl may not be suitable for standalone biofilm eradication, it could play a role when used in conjunction with other methods [57,62]. The challenge lies in reconciling laboratory results with the complexities of human responses and the realities of surgical environments.

Patient compliance with antimicrobial protocols is another vital factor influencing the success of these strategies. Preclinical studies have established the importance of education and adherence to medication regimens in reducing infection risks; however, noncompliance continues to double the risk of postoperative infections following breast surgery [155]. Therefore, integrating patient education into the clinical framework is essential for translating preclinical findings into successful outcomes.

In conclusion, while preclinical research has provided invaluable insights into effective antimicrobial strategies, translating these findings into clinical applications within breast implant surgery requires the establishment of evidence-based guidelines and standardized practices. Addressing the existing gaps between research and clinical application is paramount to optimize patient outcomes and minimize the incidence of complications related to bacterial contamination.

## 5. Advances in Implant-Shell-Material and Non-Pharmacological Strategies to Prevent Biofilm-Associated Fibrosis

Fibrotic capsule formation around implanted biomaterials, including breast implants, is a significant clinical issue associated with biofilm formation and microbial colonization. This chapter reviews non-pharmacological strategies that target microbial inhibition and biofilm prevention to mitigate fibrotic responses from a molecular and immunological standpoint.

### 5.1. Surface Characteristics and Their Influence on Biofilm Dynamics

#### 5.1.1. Surface Modification

Surface roughness of SMIs plays a significant role in biofilm formation [106,119]. Implants with rougher surfaces (Ra 60 µm) have been shown to not only attract more bacteria but also support the development of thicker and more mature biofilms compared to smoother implants (Ra 4 µm) [50]. The increased surface area and micro-topographical features of rough implants provide niches that protect bacteria from shear forces and immune surveillance, allowing them to establish robust biofilms [167].

In vitro studies have further demonstrated that the surface texture of SMIs directly influences bacterial behavior. Both *Staphylococcus aureus* and *S. epidermidis*, common culprits in implant-related infections, show increased adhesion and biofilm formation on rougher SMI surfaces [50,167]. This was corroborated by electron microscopy, which reveals that biofilms on rough surfaces are more complex and structured than those on smoother surfaces [50,54,55,57,125].

The implications of this are significant for clinical outcomes. Rougher SMIs are more likely to harbor biofilms, which can lead to persistent infections, chronic inflammation, and increased rates of capsular contracture [48,59,106,168]. Moreover, the association between textured implants and breast implant-associated anaplastic large cell lymphoma (BIA-ALCL) is thought to be related to the enhanced likelihood of biofilm formation on these surfaces, which may trigger chronic immune responses and, in some cases, contribute to tumorigenesis [48,168].

#### 5.1.2. Polyurethan Coatings

Early efforts to modify implant surfaces involved the application of polyurethane foam coatings, which were designed to disrupt the formation of fibrotic tissue by altering the implant’s surface characteristics [169,170,171]. Although initial studies indicated a reduction in fibrotic capsule formation, the widespread use of these coatings was curtailed due to concerns over the release of degradation products such as 2,4-toluene diamine (TDA), which raised potential toxicity issues [172]. Despite these concerns, newer iterations of polyurethane-coated implants are being investigated for their efficacy in controlling microbial adhesion and preventing biofilm formation. [170,171,173].

In a recent clinical study, Catanuto and colleagues [174] demonstrated that contemporary polyurethane-coated devices significantly reduced the incidence of complications such as capsular contracture and bacterial infections when compared to silicone-based implants. This reduction is attributed to the modification of the implant surface, which effectively limits bacterial adhesion—a key factor in biofilm development and fibrosis. Furthermore, despite the initial concerns regarding the toxicity of degradation products like TDA, newer generations of polyurethane implants have exhibited improved safety profiles, with no significant long-term toxicity observed in clinical trials [174,175]. These advancements underscore the critical role of polyurethane coatings in reducing microbial colonization and biofilm-associated complications, further enhancing the clinical outcomes of implant-based procedures.

### 5.2. Advances in Coating Technologies to Prevent Bacterial Adhesion

Efforts to mitigate biofilm formation on silicone breast implants have expanded with the development of antiadhesive polymeric coatings, such as hydrophilic polymer brush-based coatings. These coatings, initially explored for other medical devices, exhibit significant potential for reducing bacterial adhesion on silicone-based materials. Hydrophilic polymer brushes are non-fouling and resistant to protein adhesion, making them an ideal candidate for preventing bacterial colonization [176,177,178,179].

#### 5.2.1. Antiadhesive Coatings

In recent studies, zwitterionic coatings applied through polydopamine-assisted deposition have demonstrated a significant reduction in bacterial adhesion, with over 95% efficacy in the short term and maintaining over 90% in longer-term assessments [178]. Similar approaches using multi-arm star copolymers have shown excellent resistance to protein deposition, with efficiency levels of 96–99% compared to untreated surfaces [180]. These coatings, though originally developed for other applications, offer promising potential for use on silicone implants to limit microbial attachment and biofilm formation, potentially lowering the incidence of infections and complications such as capsular contracture.

In addition to polymer brushes, anti-adhesive hydrogels have exhibited strong resistance to protein adsorption and biofilm formation against multiple bacterial strains, including *S. aureus* and *E. coli* [181,182,183]. In vitro studies have demonstrated that certain hydrogels can completely inhibit bacterial attachment in early-stage evaluations [181]. These advancements suggest that integrating hydrogels with silicone implants could be an effective strategy to reduce biofilm-related complications in clinical settings.

#### 5.2.2. Nanoparticles

Nanoparticles have garnered attention for their ability to combat bacterial adhesion [184,185,186,187,188]. Metal oxide nanoparticles (MO-NPs) are used as great agents to catalyze the production of toxic species, including reactive oxygen species (ROS), by inducing oxidative stress [189,190]. For example, silver nanoparticles (AgNPs) are known for their antimicrobial properties, particularly through interactions with thiol groups in bacterial compounds [191,192]. Research has demonstrated that AgNP-coated silicone materials can significantly reduce biofilm formation while maintaining biocompatibility with fibroblasts [193,194,195]. Metal oxide nanoparticles, such as zinc oxide (ZnO) [196,197,198] and titanium dioxide (TiO2) [186,188,198], have also been explored for their broad-spectrum antimicrobial properties. Recent developments have led to the use of glyco-combined nanoparticles, which exhibit enhanced antibacterial effects due to their ability to penetrate bacterial membranes. For instance, silver nanoparticles associated with glycoproteins, Kucoria rosea exopolysaccharides [199], have shown significant efficacy against *S. aureus* and *E. coli*, while maintaining the biocompatibility of silicone surfaces. Furthermore, copper-coated polymer films fabricated through aerosol-assisted chemical vapor deposition (AACVD) demonstrate strong antibacterial properties, achieving a 4-log reduction in bacterial viability against both *E. coli* and *S. aureus* [200]. Additionally, the enhanced surface wettability of copper-coated films supports excellent cell adhesion and proliferation [200], indicating a dual functionality that benefits both antibacterial action and cellular interactions.

#### 5.2.3. Biomimetic Coatings

Collagenization plays a critical role in the encapsulation of foreign materials during the inflammatory response, making the strategic application of collagen coatings a promising approach for preventing biofilm formation while enhancing biocompatibility [201]. Functionalization of polydimethylsiloxane (PDMS) surfaces with acrylic acid using argon plasma activation followed by the immobilization of polyethylene glycol (PEG) chains bearing amine groups has been shown to significantly enhance cellular interactions. This method facilitates collagen immobilization via flexible spacers, resulting in improved adhesion and proliferation of fibroblast cells. Notably, the optimal PEG chain length of 6000 Da has been identified as promoting enhanced fibroblast adhesion, thus supporting tissue integration [201]. Furthermore, plasma-mediated collagen-I coatings applied to silicone implants via glow-discharge plasma treatment have demonstrated increased cell affinity and biocompatibility [202]. In vitro assessments reveal that collagen coatings lead to significant improvements in both cell adhesion and viability, emphasizing the necessity of effective cellular interactions for the successful integration of implants [201,202].

In addition to collagen-based strategies, nitric oxide (NO)-releasing xerogel coatings produced through the sol-gel method exhibit notable bactericidal activity on medical-grade silicone surfaces [203]. Quantitative and qualitative analyses of infections caused by *S. aureus* indicate an 82% reduction in infection rates for coated implants compared to uncoated controls. Histological evaluations of the tissue surrounding NO-releasing implants reveal a foreign body reaction comparable to that observed with uninfected implants, characterized by enhanced vascularization [203]. These findings suggest that NO-releasing coatings have significant potential to reduce biomaterial-associated infections, thereby improving the safety and efficacy of silicone implants.

Graphene, a single-layer carbon material, has garnered considerable interest for its potential antibacterial properties. Its high surface area, excellent electrical conductivity, and unique catalytic characteristics render it suitable for various biomedical applications [204]. Oxidized graphene oxide (GO) has been identified as particularly effective against *E. coli*, attributed to its capability to induce oxidative stress and compromise bacterial membrane integrity [205]. Coatings of graphene nano-platelets (GNPs) applied to silicone surfaces have been shown to enhance antibacterial efficacy, with oxidized GNP exhibiting superior bactericidal effects compared to their non-oxidized counterparts [206]. However, while increasing the concentration of GNP can improve surface coverage, it may also inadvertently promote bacterial adhesion, highlighting the importance of optimizing concentration to balance antibacterial performance and cellular interactions.

Studies have highlighted the potential of recombinant spider silk protein eADF4(C16) as a coating for silicone implants, significantly enhancing their immuno-biocompatibility [207,208]. Silk coatings have demonstrated reduced adhesion of fibroblasts and other human cell lines, while effectively inhibiting biofilm formation by antibiotic-resistant bacteria [207,208]. In vivo experiments have shown that these coatings reduce capsule thickness and postoperative inflammation, as well as modulate the extracellular matrix and factors associated with contracture, addressing major complications of silicone-based implants [209,210]. Overall, spider silk coatings present a promising advancement in improving the safety and efficacy of medical implants.

The advancements in non-pharmacological strategies, particularly antiadhesive coatings and surface modifications, represent significant progress in addressing the challenges of biofilm formation on silicone breast implants. By minimizing bacterial adhesion and promoting biocompatibility, these innovative approaches could substantially reduce the incidence of infections and complications associated with silicone breast implants, ultimately enhancing patient outcomes.

## 6. Pharmacological Strategies to Prevent Biofilm-Associated Fibrosis in Implants

Biofilm-associated fibrosis around implants is a significant clinical challenge, primarily due to microbial infections and the subsequent fibrotic response. This chapter reviews pharmacological strategies with antimicrobial properties aimed at mitigating biofilm-associated fibrosis around implants, focusing on mechanisms of action, drug classes, and specific agents demonstrating efficacy.

### 6.1. Antibacterial Drugs

Antibacterial drugs play a crucial role in preventing biofilm formation and associated fibrosis. These drugs can be administered in various forms, including systemic, topical, or incorporated into the implant material itself.

#### 6.1.1. Systemic Antibacterials

Systemic antibiotics such as cefazolin and gentamicin are commonly used as prophylactic measures before and during surgery. While cefazolin, a first-generation cephalosporin, is recognized for its effectiveness in reducing infections associated with breast implants [211,212], gentamicin, an aminoglycoside, also demonstrates broad-spectrum activity against both Gram-positive and Gram-negative bacteria, including those commonly involved in implant infections [211,212]. However, the overall impact of these antibiotics on surgical site infection (SSI) rates continues to be a subject of debate in the literature.

#### 6.1.2. Topical Antibacterials

Topical antibiotics, including bacitracin and chlorhexidine gluconate, are applied directly to the implant or the surgical site. Bacitracin, a peptide antibiotic, is effective against Gram-positive bacteria and is used in irrigation solutions to reduce bacterial load [211,213,214,215]. Chlorhexidine gluconate, an antiseptic with broad-spectrum antimicrobial activity, has shown efficacy in reducing microbial contamination and biofilm formation on implant surfaces [216].

#### 6.1.3. Drug Incorporation into Implants

Incorporating antibiotics into the implant material itself can provide sustained antimicrobial effects. Rifampin, a macrocyclic antibiotic, has been incorporated into implant surfaces, showing effectiveness in reducing bacterial colonization and subsequent fibrosis [217]. This approach allows for localized drug delivery, potentially reducing the need for systemic antibiotics and minimizing adverse effects.

### 6.2. Antifibrotic and Anti-Inflammatory Drugs

Fibrosis, a major consequence of chronic inflammation and infection, can be targeted with antifibrotic and anti-inflammatory drugs.

#### 6.2.1. Pirfenidone

Pirfenidone is an antifibrotic drug with established efficacy in treating idiopathic pulmonary fibrosis. It works by reducing the production of inflammatory mediators and fibroblast proliferation. In preclinical models, pirfenidone administration reduced capsule thickness and fibroblast proliferation around implants [218,219,220,221]. This drug may prove beneficial in mitigating fibrotic responses associated with biofilm infections.

#### 6.2.2. Halofuginone

Halofuginone, originally developed for veterinary use, inhibits collagen type I synthesis and the differentiation of T helper 17 cells. When grafted onto the surface of silicone implants, halofuginone significantly decreased capsule thickness and FBR in animal models [222,223,224,225]. This agent’s antifibrotic properties make it a promising candidate for controlling fibrosis related to biofilm infections [226].

#### 6.2.3. Dexamethasone

Dexamethasone, a potent glucocorticoid, reduces inflammation and collagen production by modulating cytokine activity [227]. Its application in implant surgery has been shown to decrease fibrous tissue formation [213,227,228]. The intravenous administration of dexamethasone during the perioperative period may help regulate inflammation and fibrosis surrounding implants, potentially enhancing outcomes for patients susceptible to biofilm-associated fibrosis [213].

### 6.3. Integration of Antimicrobial Strategies

Effective management of biofilm-associated fibrosis often requires a combination of antimicrobial and antifibrotic approaches. For example, preoperative prophylactic antibiotics combined with local antiseptic irrigation can significantly reduce the risk of infection and biofilm formation [226]. Furthermore, while there is potential in incorporating antifibrotic agents into the implant material or using them adjunctively with antimicrobial strategies to enhance overall efficacy in preventing fibrosis, it is important to note that these strategies have primarily been demonstrated in preclinical and animal studies. There is currently insufficient evidence to support their routine application in clinical practice [226].

To address biofilm-associated fibrosis around SMI, significant progress has been made through both material-based and pharmacological strategies. Non-pharmacological approaches, including physical modifications with antimicrobial properties (such as polyurethane coatings and surface topography adjustments) and innovative biological matrices (like antibiotic-impregnated meshes and spider silk-based materials), focus on preventing microbial adhesion and biofilm formation. Concurrently, pharmacological interventions involving systemic and topical antibacterials, as well as antifibrotic agents, provide targeted solutions to manage both infection and fibrosis. The integration of these strategies, as detailed in Table 2, underscores the multifaceted approach needed to effectively mitigate fibrotic responses and improve clinical outcomes related to implant-associated complications.

### 6.4. Long-Term Effects of Antimicrobial Strategies in Implant Surgery

The incorporation of antimicrobial agents into implant materials offers significant advantages in reducing the incidence of biofilm formation and associated complications. However, the long-term implications of using antimicrobial-impregnated materials warrant careful consideration. While these strategies have demonstrated effectiveness in short-term studies, the potential risks and consequences associated with prolonged exposure to antimicrobial agents must be addressed.

One primary concern associated with the long-term use of antimicrobial agents is the potential for the development of antimicrobial resistance (AMR). Continuous exposure to subtherapeutic levels of antibiotics can select for resistant strains of bacteria, diminishing the effectiveness of these agents over time [53]. In the context of implant surgery, the emergence of resistant pathogens could lead to increased surgical site infections (SSIs), complicating patient outcomes and necessitating alternative, often more aggressive, treatment strategies.

The biocompatibility of antimicrobial-impregnated materials is crucial for their long-term use in clinical settings. Chronic exposure to certain antimicrobial agents may trigger adverse tissue reactions, including inflammation or fibrosis, which could negate the benefits of infection prevention [138]. Materials that release antimicrobial agents over extended periods may provoke ongoing inflammatory responses, leading to capsule formation or other fibrotic complications around the implant [5].

The long-term use of antimicrobial strategies can also disrupt the host microbiome, resulting in dysbiosis, which can have systemic implications for health [5]. Disruption of the normal microbial flora may increase susceptibility to opportunistic infections and alter immune responses [229]. This shift in the microbiome could affect patient recovery and the overall success of implant surgeries.

Given these potential risks, regulatory bodies emphasize the importance of thorough long-term studies to assess the safety and efficacy of antimicrobial strategies in implant surgery. Clinical trials should monitor not only the immediate outcomes but also the long-term effects of antimicrobial-impregnated materials on patient health, the development of resistance, and overall implant longevity.

While the integration of antimicrobial strategies in implant surgery presents promising avenues for reducing complications associated with biofilm formation, a balanced understanding of their long-term effects is essential. Ongoing research and clinical vigilance are necessary to ensure that the benefits of these strategies are not outweighed by their potential risks, thus safeguarding patient health and improving surgical outcomes.

## 7. Clinical Implications and Future Directions

The clinical implications of microbial adhesion, colonization, and biofilm formation on SMIs are extensive, influencing both patient outcomes and the effectiveness of treatment strategies. Understanding these processes from a molecular and immunological perspective is crucial for developing improved preventive and therapeutic measures.

Biofilm formation on SMIs is a significant concern due to its impact on implant longevity and patient health. Biofilms, which consist of microorganisms embedded in a self-produced extracellular matrix, offer protection from both host immune responses and antimicrobial treatments [54,125]. This persistent biofilm can lead to chronic inflammation and fibrosis, complicating implant-related conditions. Persistent biofilms exacerbate inflammatory responses, as the biofilm matrix hinders immune cell infiltration and the effectiveness of antimicrobial peptides [54,55,125]. This chronic inflammation often results in a thick fibrous capsule around the implant, potentially leading to complications such as chronic pain, impaired function, and implant failure [53,55,58].

Current strategies to manage these issues include preoperative antimicrobial prophylaxis, antimicrobial coatings, and advanced materials designed to reduce microbial adhesion and biofilm formation. However, the effectiveness of antimicrobial strategies remains limited due to the rise of antimicrobial resistance and variability in clinical practices [57,59,116]. The challenge of biofilm-associated infections underscores the need for more innovative and effective approaches to prevent and manage these complications.

Recent advances in material science and pharmacology offer promising solutions. Antibiotic-impregnated meshes and spider silk-based meshes represent significant progress in preventing microbial adhesion and biofilm formation [207,230]. These materials provide sustained antimicrobial activity or reduce bacterial adhesion through physical and chemical modifications. Additionally, zwitterionic polymers, known for their resistance to protein adsorption and microbial colonization, show potential in mitigating fibrotic responses [231,232]. The integration of antifibrotic and anti-inflammatory agents, such as pirfenidone, halofuginone, and dexamethasone, either incorporated into implant materials or used as adjunctive therapies, further enhances the management of biofilm-associated complications [218,222,227]. These agents can modulate the inflammatory response and reduce fibrosis, offering a targeted approach to managing complications.

Looking forward, several key areas warrant further exploration. Developing targeted antimicrobial strategies that effectively penetrate and disrupt biofilms is crucial. Novel drug delivery systems, such as nanoparticles or localized drug reservoirs, could enhance the efficacy of antimicrobial treatments [217]. Additionally, exploring biomimetic and smart materials that can dynamically respond to microbial threats represents an exciting frontier. These materials could release antimicrobial agents in response to biofilm formation or alter their properties to prevent microbial adhesion [207,232].

Personalized medicine approaches also hold significant promise. Tailoring antimicrobial and antifibrotic treatments based on individual patient factors, such as microbial flora, immune responses, and genetic predispositions, could greatly improve clinical outcomes [218,222]. Moreover, establishing standardized clinical guidelines for antimicrobial prophylaxis, implant coatings, and antifibrotic treatments is essential. Such guidelines would help ensure consistent and effective management of biofilm-associated complications across clinical settings. Ongoing research and clinical trials will be instrumental in developing these guidelines and optimizing patient care [57,59,116].

In summary (Table 3), while advances in understanding microbial contamination and biofilm formation on SMIs have led to improved management strategies, there remains a need for continued innovation and research. By focusing on targeted antimicrobial strategies, exploring novel materials, and standardizing clinical practices, we can enhance patient outcomes and address the challenges associated with biofilm formation and fibrosis.

## 8. Conclusions

The exploration of microbial adhesion, colonization, and biofilm formation on SMIs highlights a critical area of concern in both the molecular and clinical realms. The persistent challenge of biofilm-associated infections underscores the complexity of managing implant-related complications and the need for continued innovation in both material science and therapeutic strategies.

Biofilm formation on SMIs represents a multifaceted issue, with significant implications for patient health and implant longevity. The inherent protective properties of biofilms, which shield bacteria from both immune responses and antimicrobial treatments, contribute to the chronic nature of infections and the difficulty of eradicating these microbial communities. The extracellular polymeric substances (EPSs) that form part of the biofilm matrix play a crucial role in this persistence, effectively hindering the penetration of immune cells and antimicrobial agents. This results in prolonged inflammation and fibrosis, complicating implant-related conditions and impacting patient outcomes.

Recent advancements in material science, such as the development of antimicrobial coatings, antibiotic-impregnated meshes, and spider silk-based materials, offer promising solutions to mitigate biofilm formation and reduce microbial colonization. However, the manufacturing processes of these novel materials may pose practical limitations and challenges, including scalability, cost, and the complexity of regulatory approvals, which need to be addressed to facilitate their clinical application. These innovations aim to enhance the effectiveness of SMIs by either preventing bacterial adhesion or providing sustained antimicrobial activity. Zwitterionic polymers, with their resistance to protein fouling, represent another promising avenue for reducing microbial colonization and associated fibrosis. Incorporating antifibrotic and anti-inflammatory agents into implant materials or using them as adjunctive therapies further supports the management of biofilm-associated complications, providing targeted approaches to control inflammation and fibrosis.

Despite these advancements, several challenges remain. The rise of antimicrobial resistance (AMR) is a significant concern, as continuous exposure to subtherapeutic levels of antimicrobial agents can select for resistant bacterial strains. This highlights the need for further research into alternative antimicrobial strategies and innovative drug delivery systems that can minimize resistance development while effectively managing infections. The variability in clinical practices underscore the need for more effective and standardized approaches to antimicrobial prophylaxis and implant management. The development of targeted drug delivery systems and personalized medicine strategies could significantly improve the efficacy of treatments by tailoring interventions to individual patient factors and specific microbial threats. Establishing evidence-based guidelines for antimicrobial prophylaxis, implant coatings, and antifibrotic treatments is essential for ensuring consistent and effective management across clinical settings.

Future research should focus on several key areas: enhancing our understanding of biofilm dynamics and microbial interactions with implant surfaces, developing innovative materials that dynamically respond to microbial threats, and optimizing personalized treatment strategies. In particular, interdisciplinary collaboration among material scientists, microbiologists, and clinicians will be crucial to drive forward the development of new solutions tailored to combat biofilm formation effectively. Continued investigation into novel materials and therapeutic approaches will be instrumental in addressing the challenges associated with biofilm formation and fibrosis. Clinical trials and ongoing research will play a crucial role in refining these strategies and improving patient outcomes.

In conclusion, while significant progress has been made in understanding and addressing microbial contamination and biofilm formation on SMIs, there remains a critical need for continued innovation and research. By focusing on targeted antimicrobial strategies, exploring advanced materials, and establishing standardized clinical practices, we can improve the management of implant-related complications and enhance patient care. Furthermore, a balanced understanding of both the short- and long-term effects of antimicrobial strategies is essential to ensure patient safety and optimize surgical outcomes. The integration of molecular insights with practical clinical approaches will be key to overcoming the challenges posed by biofilm-associated infections and ensuring the long-term success of medical implants.

## Figures and Tables

**Table 1:** Key physiological processes and their impact on wound healing around silicone mammary implants.

Biological Mechanisms	Wound Healing Dynamics	Fibrotic Responses in SMI
**Pathophysiological Process**	Restoration of tissue integrity and function through hemostasis, inflammation, proliferation, and remodeling.	Formation of a fibrous capsule around implants due to inflammatory response and biofilm formation.
**Initial Response**	Hemostasis: Constriction of blood vessels, platelet activation, clot formation to prevent blood loss and provide a scaffold for healing.	Immediate Immune Response: Activation of innate immune system, recruitment of immune cells (neutrophils, macrophages) due to microbial contamination and protein adsorption.
**Molecular Components**	Platelets, Fibrinogen, Fibrin: Form a clot that acts as a barrier and temporary scaffold.	Microorganisms, Proteins: Microbial biofilm formation on the implant surface, leading to persistent immune stimulation.
**Inflammatory Phase**	Neutrophils and Macrophages: Clear debris, combat infection, release cytokines and MMPs to modulate the inflammatory response.	Chronic Inflammation: Persistent inflammation due to biofilm, leading to prolonged immune response and ongoing ECM remodeling.
**Proliferation Phase**	Fibroblast Proliferation, ECM Deposition, Angiogenesis, Epithelialization: Formation of new tissue, collagen production, and blood vessel formation.	Fibrous Capsule Formation: Collagen and ECM deposition around the implant, leading to capsule formation.
**Remodeling Phase**	Collagen Realignment, Cross-Linking, Continual ECM Remodeling: Strengthening of new tissue and refinement of the wound architecture.	Capsular Contracture: Excessive collagen deposition and ECM remodeling leading to a contracted and thickened fibrous capsule.
**Impact on Healing**	Prevention of Infection, Scar Formation: Effective healing reduces infection risk, minimizes scar formation, and restores tissue function.	Infection Risk: Biofilms create a persistent infection risk that complicates healing and exacerbates fibrosis.

**Table 2:** Non-pharmacological and pharmacological strategies to prevent biofilm-associated fibrosis in implants.

Category	Strategy	Mechanism	Key Points
**Physical** **Modifications with** **Antimicrobial** **Properties**	**Polyurethane Foam Coatings**	Surface modification to disrupt fibrotic tissue formation	Initial reduction in fibrotic capsule formation, concerns over toxicity from degradation products [168,169,170,171,172].
	**Surface** **Topography**	Rough surfaces increase biofilm formation	Rougher surfaces (Ra 60 µm) enhance bacterial adhesion and biofilm maturity, leading to infections and capsular contracture [45,47,105,174].
**Biological** **Matrices with** **Antimicrobial** **Properties**	**Antibiotic-** **Impregnated Meshes**	Localized antibiotic delivery to reduce biofilm formation	Promising in reducing bacterial colonization and biofilm formation with sustained antimicrobial activity [175,176,177,178].
	**Spider Silk-** **Based Meshes**	Inhibits bacterial adhesion and fibrotic tissue formation	Biocompatible, reduces fibroblast proliferation and collagen deposition [179,180,181,182,183,184,185].
	**Zwitterionic** **Polymers**	Superior hydrophilicity, preventing protein adsorption and microbial adhesion	Resistant to microbial colonization, preventing foreign body response and fibrotic capsules [186,187,188,189,190].
**Pharmacological Strategies**	**Systemic** **Antibacterials (Cefazolin,** **Gentamicin)**	Prophylactic antibiotics to prevent infections before and during surgery	Effective against Gram-positive and Gram-negative bacteria in breast implant surgeries [191,192].
	**Topical** **Antibacterials (Bacitracin,** **Chlorhexidine)**	Direct application to reduce bacterial load and biofilm formation	Applied during surgery to prevent contamination and infection [191,193,194,195].
	**Drug** **Incorporation into Implants** **(Rifampin)**	Localized, sustained antimicrobial effect from drug-coated implant surfaces	Reduces bacterial colonization and biofilm formation directly at the implant site [196].
**Antifibrotic and Anti-inflammatory Drugs**	**Pirfenidone**	Reduces inflammation and fibroblast activity	Shown to reduce capsule thickness in preclinical models, potential use in biofilm-associated fibrosis [197,198,199,200].
	**Halofuginone**	Inhibits collagen synthesis and T helper 17 cell differentiation	Reduces fibrosis and capsule formation around implants, promising antifibrotic properties [201,202,203,204].
	**Dexamethasone**	Reduces inflammation and collagen production by modulating cytokine activity	Decreases fibrous tissue formation and inflammation, improving implant surgery outcomes [193,206,207].
**Integration of** **Antimicrobial** **and Antifibrotic Strategies**	**Combined** **Approaches**	Use of both antimicrobial and antifibrotic strategies to prevent biofilm formation and fibrosis	Combining antibiotics with local antiseptic irrigation and antifibrotic agents enhances efficacy in preventing biofilm-associated fibrosis.

**Table 3:** Challenges, current strategies, and future directions for improving the clinical management of biofilm-associated complications in SMI-based breast surgery.

Clinical Aspect	Challenges	Current Strategies	Future Directions
**Biofilm** **Formation** **on SMIs**	Persistent biofilms protect bacteria from immune responses and antimicrobials, leading to chronic inflammation and fibrosis.	Antimicrobial prophylaxis, antimicrobial coatings, and advanced material surfaces designed to reduce biofilm formation.	Develop targeted antimicrobial strategies that penetrate biofilms. Biomimetic materials that release antimicrobials in response to biofilm formation.
**Chronic** **Inflammation and Fibrosis**	Biofilm matrix limits immune cell infiltration and antimicrobial effectiveness, leading to thick fibrous capsules.	Anti-inflammatory and antifibrotic agents (pirfenidone, halofuginone, dexamethasone) to reduce fibrosis.	Incorporate anti-inflammatory and antifibrotic agents into implant materials. Personalized treatment approaches based on patient immune responses.
**Antimicrobial Resistance**	Resistance to traditional antimicrobial treatments is rising, making it difficult to manage biofilm infections.	Preoperative prophylaxis with systemic antibiotics; antimicrobial materials such as antibiotic-impregnated meshes.	Novel drug delivery systems (e.g., nanoparticles or localized reservoirs) that enhance antimicrobial efficacy and overcome resistance.
**Advanced** **Materials**	Existing materials may not fully prevent microbial adhesion or fibrosis.	Antibiotic-impregnated and spider silk-based meshes, zwitterionic polymers.	Development of smart materials that dynamically respond to microbial threats, change surface properties, or release antimicrobials.
**Clinical** **Guidelines** **and Practice**	Variability in practices and lack of standardized guidelines lead to inconsistent management of biofilm complications.	General antimicrobial prophylaxis and surface modifications for reducing biofilm risk.	Standardized clinical guidelines for antimicrobial prophylaxis, coatings, and antifibrotic treatments across clinical settings.
**Personalized Medicine**	Generalized treatments may not consider individual patient factors, leading to suboptimal outcomes.	Uniform antibiotic and antifibrotic regimens based on general risk profiles.	Personalized treatments tailored to patient-specific factors (microbial flora, immune responses, genetic predisposition).

## Data Availability

Not applicable.
